# Integrative genomic expression analysis reveals stable differences between lung cancer and systemic sclerosis

**DOI:** 10.1186/s12885-021-07959-6

**Published:** 2021-03-10

**Authors:** Heng Li, Liping Ding, Xiaoping Hong, Yulan Chen, Rui Liao, Tingting Wang, Shuhui Meng, Zhenyou Jiang, Dongzhou Liu

**Affiliations:** 1 Department of Rheumatology and Immunology, Shenzhen People’s Hospital, The Second Clinical Medical College of Jinan University, Shenzhen, 518020 China; 2 Integrated Chinese and Western Medicine Postdoctoral Research Station, Jinan University, Guangzhou, 510632 China; 3 Department of Microbiology and Immunology, College of Basic Medicine and Public Hygiene, Jinan University, Guangzhou, 510632 China; 4 The First Affiliated Hospital (Shenzhen People’s Hospital) Southern University of Science and Technology, Shenzhen, 518055 China

**Keywords:** Lung cancer, Systemic sclerosis, Bioinformatics, Immunity

## Abstract

**Background:**

The incidence and mortality of lung cancer are the highest among all cancers. Patients with systemic sclerosis show a four-fold greater risk of lung cancer than the general population. However, the underlying mechanism remains poorly understood.

**Methods:**

The expression profiles of 355 peripheral blood samples were integratedly analyzed, including 70 cases of lung cancer, 61 cases of systemic sclerosis, and 224 healthy controls. After data normalization and cleaning, differentially expressed genes (DEGs) between disease and control were obtained and deeply analyzed by bioinformatics methods. The gene ontology (GO) and Kyoto Encyclopedia of Genes and Genomes (KEGG) pathway enrichment analysis were performed online by DAVID and KOBAS. The protein–protein interaction (PPI) networks were constructed from the STRING database.

**Results:**

From a total of 14,191 human genes, 299 and 1644 genes were identified as DEGs in systemic sclerosis and lung cancer, respectively. Among them, 64 DEGs were overlapping, including 36 co-upregulated, 10 co-downregulated, and 18 counter-regulated DEGs. Functional and enrichment analysis showed that the two diseases had common changes in immune-related genes. The expression of innate immune response and response to virus-related genes increased significantly, while the expression of negative regulation of cell cycle-related genes decreased notably. In contrast, the expression of mitophagy regulation, chromatin binding and fatty acid metabolism-related genes showed distinct trends.

**Conclusions:**

Stable differences and similarities between systemic sclerosis and lung cancer were revealed. In peripheral blood, enhanced innate immunity and weakened negative regulation of cell cycle may be the common mechanisms of the two diseases, which may be associated with the high risk of lung cancer in systemic sclerosis patients. On the other hand, the counter-regulated DEGs can be used as novelbiomarkers of pulmonary diseases. In addition, fat metabolism-related DEGs were consideredto be associated with clinical blood lipid data.

**Supplementary Information:**

The online version contains supplementary material available at 10.1186/s12885-021-07959-6.

## Background

One in six people dies of cancer worldwide and the cancer burden is increasing each year [1]. In 2018, approximately 9.6 million people worldwide died of cancer, from which lung cancer was the most commonly diagnosed cancer [1, 2]. There is some evidence that patients with autoimmune diseases have an increased risk of cancer due to immune system disorders [3, 4]. Dozen of autoimmune diseases could increase the incidence and mortality of lung cancer [5–9], but the underlying regulations of the associated genes’ expression remain unclear. The accumulated gene expression data of related diseases in recent years havebrought opportunities to provide clues of mechanism of expressional regulation.

The immune system plays different roles in combatting different diseases. Autoimmune diseases and cancers have certain correlations and differences. Both lung cancer and systemic sclerosis are associated with pulmonary fibrosis [10, 11], and the early diagnosis is challenging. In addition, pulmonary manifestations of lung cancer and systemic sclerosis are easy to be confused with each other, even with the help of radiological and histopathological evaluation. The major risk factors for lung cancer include external risk factors such as smoking, occupational exposure, air pollution, ionizing radiation and diet, as well as internal risk factors e.g. genetics and lung disease history [12, 13]. Although systemic sclerosis is a rare disease, recent studies have shown that it is linkedto an increased risk of lung cancer [14, 15]. A meta-analysis of 16 studies involving more than 7000 patients revealed that, compared with the general population, scleroderma patients had a significantly higher risk of lung cancer (RR 4.35; 95% CI 2.08, 9.09) [16]. A relationship of immune abnormalities in systemic sclerosis and lung cancer is unclear and needs to be investigated to understand the risk association between these two diseases. Although recent studies have shown certain gene signatures of systemic sclerosis or lung cancer [17–19], the accuracy and stability of the results need to be improved due to their small sample size.

Here, we conducted a comprehensive analysis to clarify the relationship of immune-related genes between systemic sclerosis and lung cancer from an intersection of 14,191 genes from four datasets (*n* > 15). Using data from international sources, we discovered evidence of common gene expressional changes between systemic sclerosis and lung cancer, and identified the corresponding biological processes. Most importantly, we discovered immune genes with opposite expressional trends in systemic sclerosis and lung cancer. Together with rich functional annotation, these findings will help revealing the development mechanisms of the two diseases.

## Methods

### Data sources and patients

DNA microarray analysis is a reliable technology for analyzing gene expression profiles. The gene expression profiling of lung cancer and systemic sclerosis whole blood samples were used in this study. For systemic sclerosis, which is a rare disease, we used the accumulated data from many years. We considered necessary to have large sample sizes to reduce batch differences (number of patients > 15) and included more healthy controls to provide a reliable reference. The accession and platform information for 61 patients with systemic sclerosis, 70 patients with lung cancer, and 224 normal healthy individuals is presented (Table S1).

Patients with systemic sclerosis from January 1, 2018 to December 1, 2020 in the Department of Rheumatology and Immunology of Shenzhen People’s Hospital was diagnosed by two or more experienced doctors according to the 2013 classification criteria for systemic sclerosis. Patients with complications were excluded. The data of healthy controls were collected from the data management platform of Shenzhen People’s Hospital and matched with each sample according to age (±1 year).

### Integration of microarray data

All packages were executedin R software (version 4.0.1). Data normalization and DEGs identification was performed using limma package (*P* value < 0.05). The RRA package was utilized to analyze common DEGs (*P* value < 0.05). Genes with up- or down-regulated expression in three chips were used in subsequent analysis. The packages are publicly available in the Comprehensive R Network. The 299 and 1644 DEGs were identified in systemic sclerosis and lung cancer, respectively, of which 64 DEGs overlapped, including 36 co-upregulated DEGs, 10 co-downregulated DEGs and 18 DEGs with opposite regulatory trends. Pheatmap package was used to generate heatmaps.

### Functional annotation and pathway mapping

DAVID Bioinformatics Resources 6.8 (https://david.ncifcrf.gov/) was employed for functional annotation of the DEGs primarily associated with either systemic sclerosis or lung cancer [20]. Pathways were enriched from these DEGs using web-based knowledge databases KEGG, GO and BioCarta [21]. KEGG pathway mapping was utilized to generate toll-like receptors signaling pathways.

### Data analysis and statistical analysis

Microsoft Excel 2013 was used to store and organize data. GraphPad Prism 5 was used to generate graphics. The DEGs for each disease were derived by comparison with sex- and age-matched controls (within 10% difference). DEG was defined as gene with fold change > 1.2 or < 0.8. Mean fold change of important genes was visualized by color intensity (*P* < 0.05), red for up-regulation, and green for down-regulation.

Statistical analysis was conducted using SPSS 17.0 software (SPSS, Chicago, USA). The t-test was applied while comparing groups. The significance level was set at *P* < 0.05.

## Results

### Improving the robustness of gene expression profiles

**Fig. 1:**
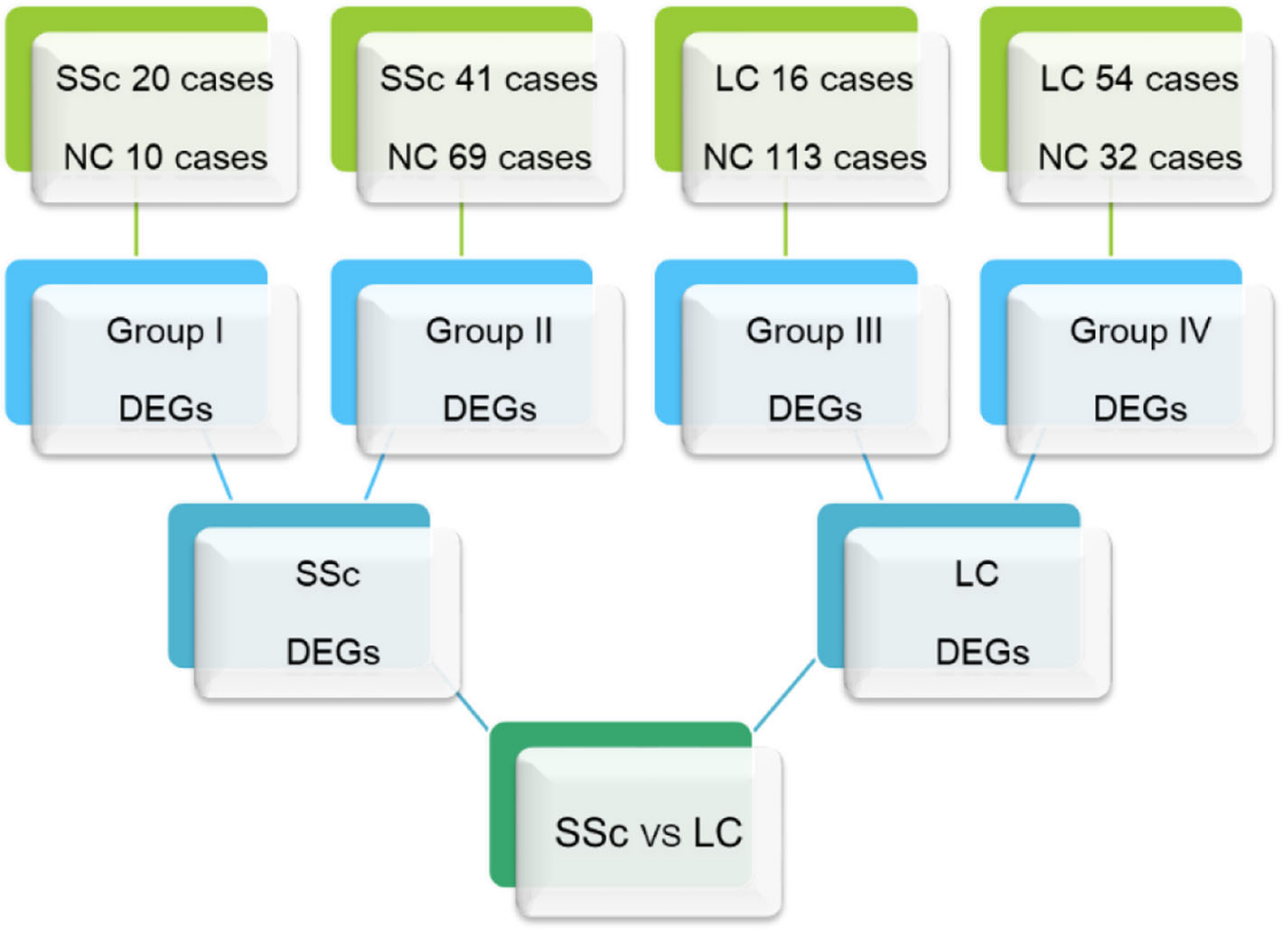
A scheme of systemic sclerosis (Group I and Group II) and lung cancer (Group III and Group IV) gene expression analysis. SSc: systemic sclerosis, LC: lung cancer, NC: normal control, DEG: differentially expressed gene

**Fig. 2:**
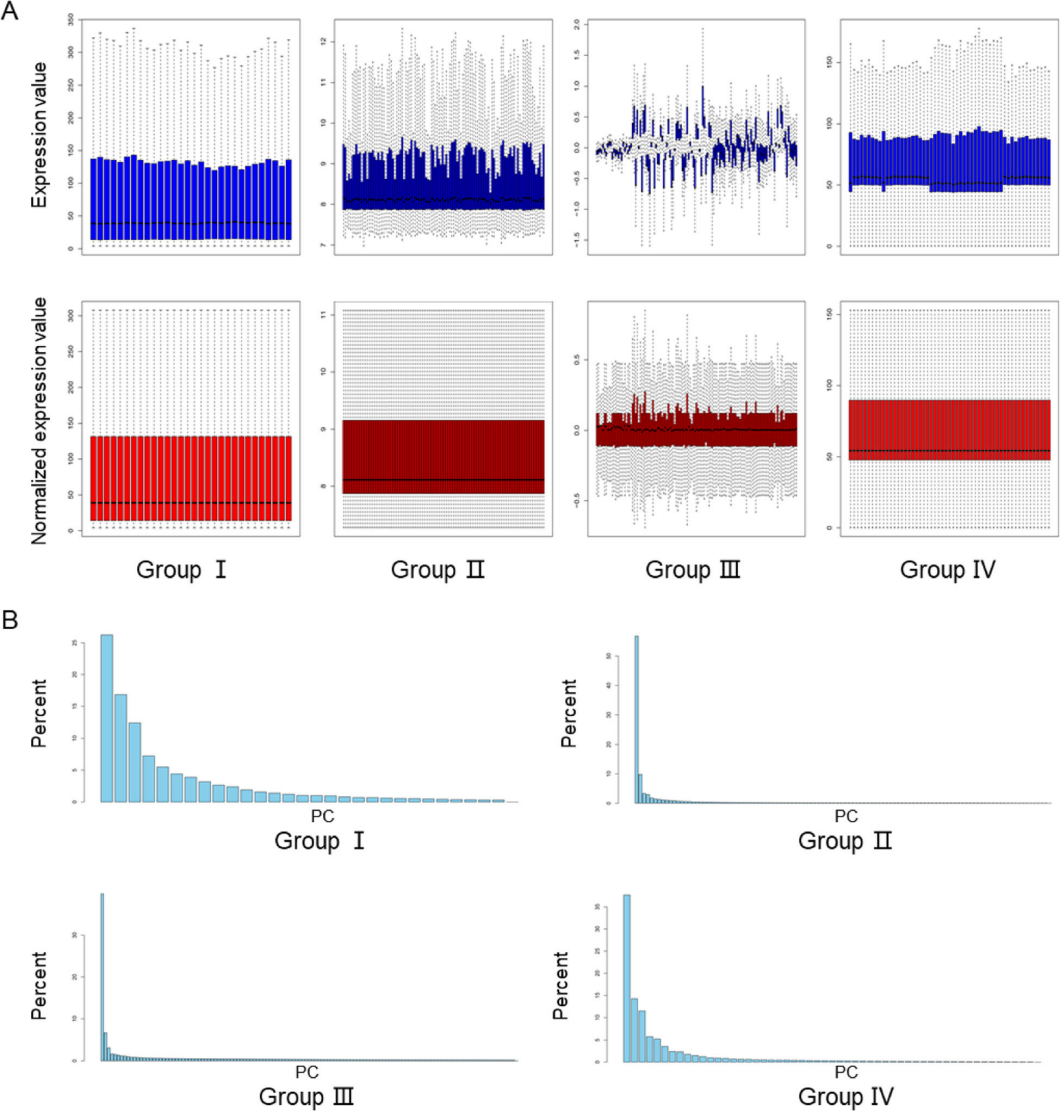
Gene expression profile data pre-processing. **a** Normalization of gene expression data. Blue bars represent data before normalization and red bars represent normalized data. **b** Principal component analysis of the standardized data

**Fig. 3:**
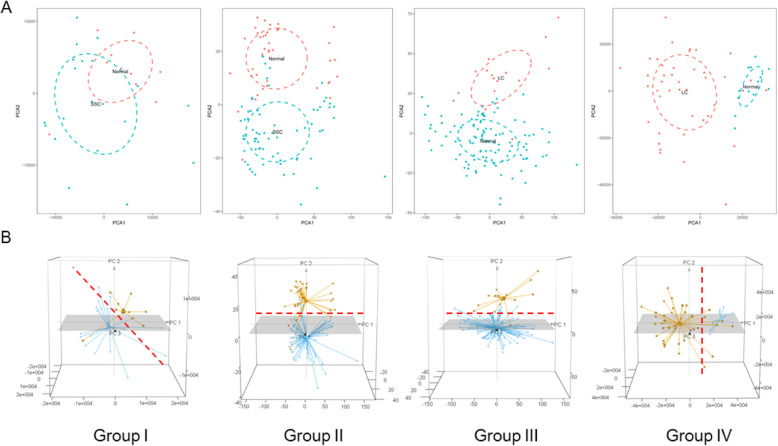
Two-dimensional (**a**) and three-dimensional (**b**) principal component analysis, showing in relationship between disease and normal samples

The whole process of a comprehensive gene expression analysis of primary systemic sclerosis or primary lung cancer is presentedin Fig. 1. At first, the disease and control data were compared for each group, and then the differences between the two diseases were analyzed. The gene expression profiles of peripheral blood from 61 patients with primary systemic sclerosis, 70 patients with primary lung cancer, and 224 normal healthy individuals were obtained from the Gene Expression Omnibus database (Table S1). The datasets with a number of patients below 15 were excluded to reduce batch differences between different experiments. The collected data were pre-processed to meet analysis requirements. To improve reliability of disease analysis, after standardization of the data (Fig. 2a), a principal component analysis was performed (Fig. 2b). All outliers outside the interface of the two diseases were removed (separated by red dotted lines in Fig. 3, listed in Table S1). The obtained gene expression data enabled us to systematically identify and mine important disease-related genes.

### Genomic expression integration and comparison

**Fig. 4:**
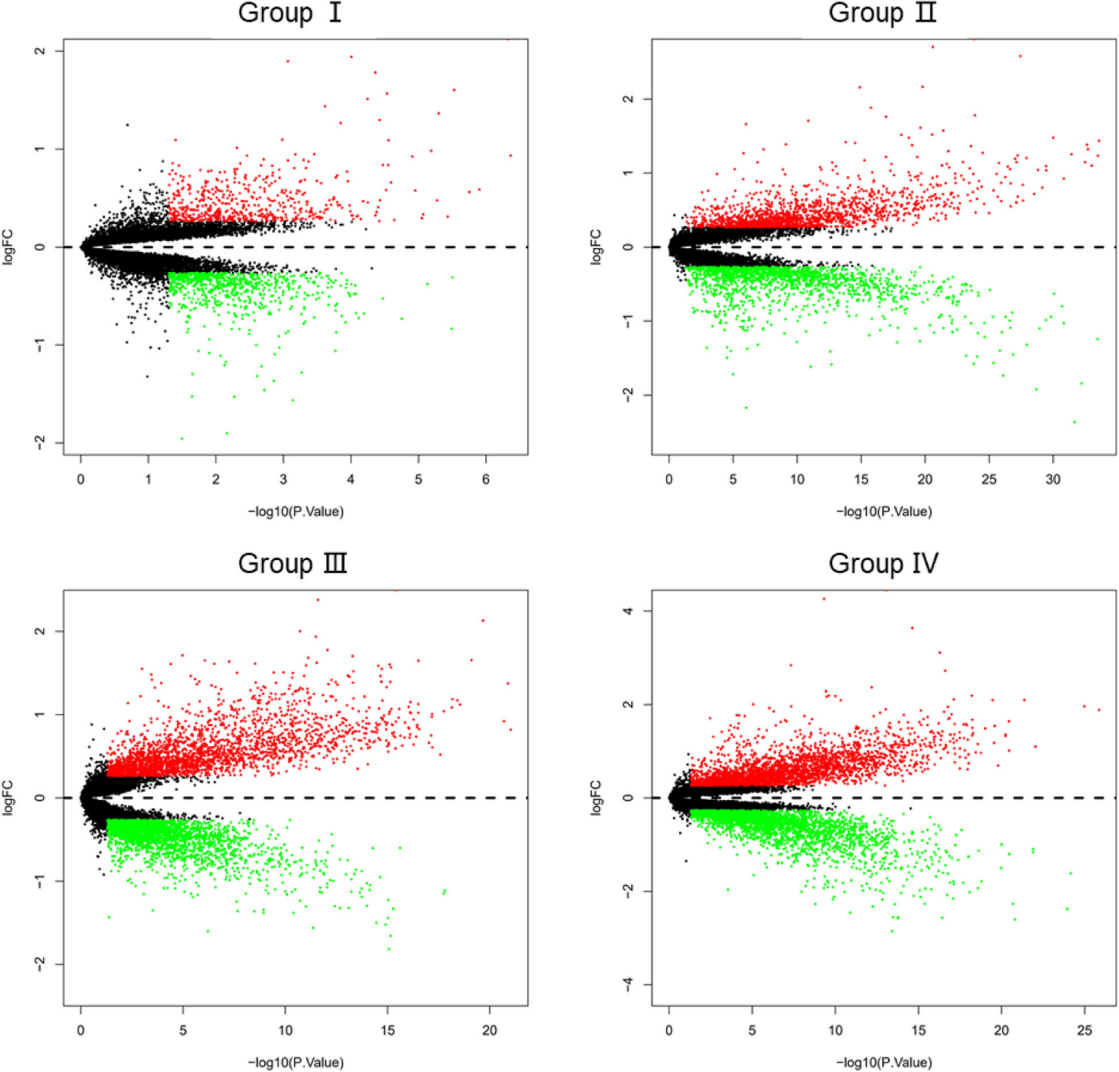
Differential expression between diseases and healthy controls (fold change > 1.20/ fold change < 0.80 and *P*-value < 0.05). The red and green points represent up- and down-regulated genes, respectively. The black points represent genes with no significant difference. FC: fold change

**Fig. 5:**
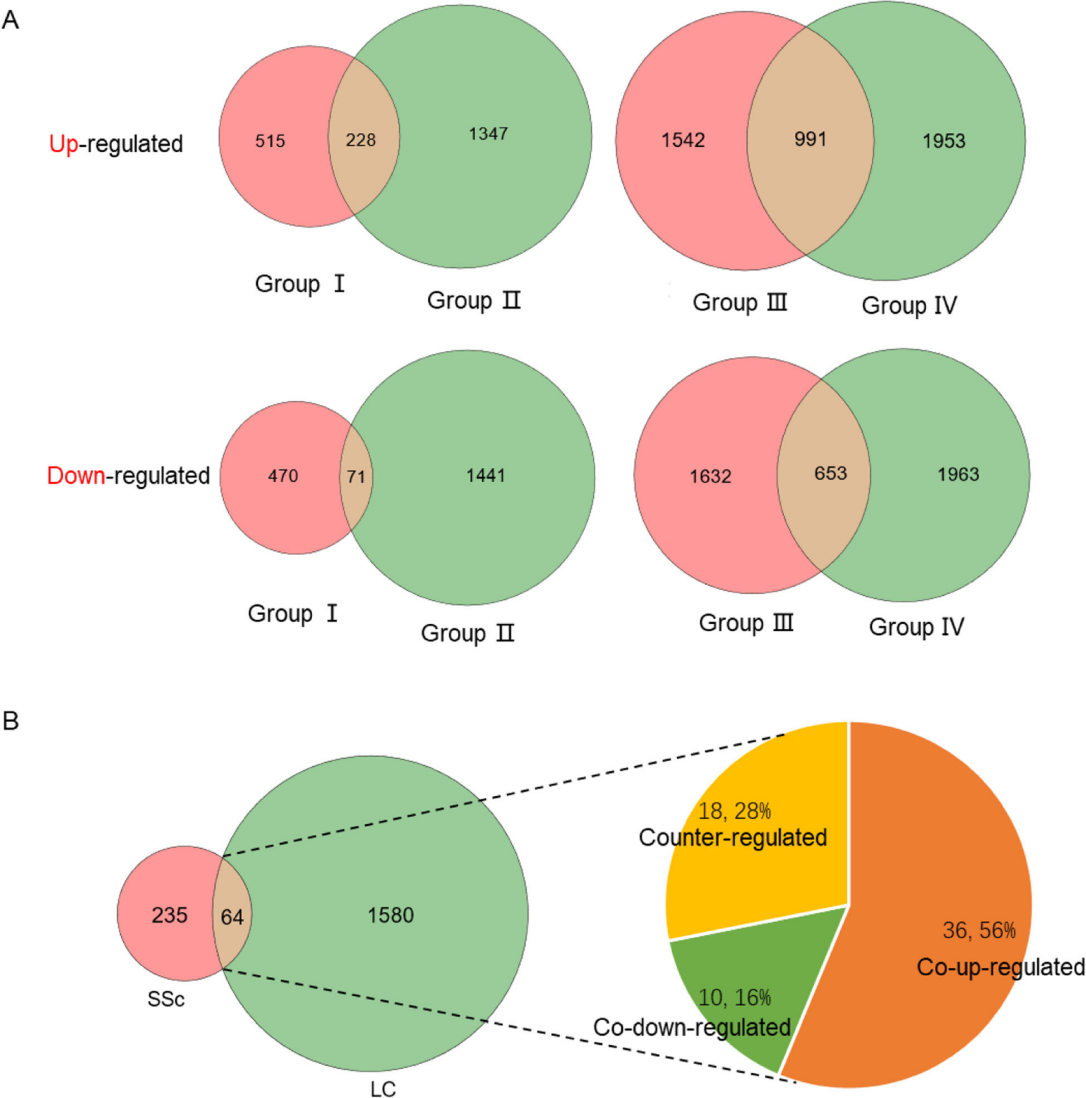
**a** Co-upregulated and co-downregulated genes of systemic sclerosis (Group I and Group II) and lung cancer (Group III and Group IV). **b** The overlapping DEGs in systemic sclerosis and lung cancer including up-, down- and counter-regulated genes

Based on the principal component analysis, we examined DEGs identified in four groups screened by the limma package (fold change > 1.20/ fold change < 0.80 and *P*-value < 0.05) (Fig. 4), and performed a cluster analysis of the top-ranked genes (Fig. S1). The number of upregulated genes was equal to that of downregulated genes in each set of data. To improve the reliability of identification of disease-related genes, the DEGs of each group were analyzed and integrated into co-upregulated or co-downregulated genes according to different disease types (Fig. 5a). Overall, 299 DEGs were screened from the systemic sclerosis data (Group I and Group II), including 228 up-regulated genes and 71 down-regulated genes. Additionally, 1644 DEGs were screened from the lung cancer data, including 991 upregulated genes and 653 downregulated genes (Group III and Group IV). The results showed that DEGs in lung cancer were about five times as those DEGs in systemic sclerosis.

**Fig. 6:**
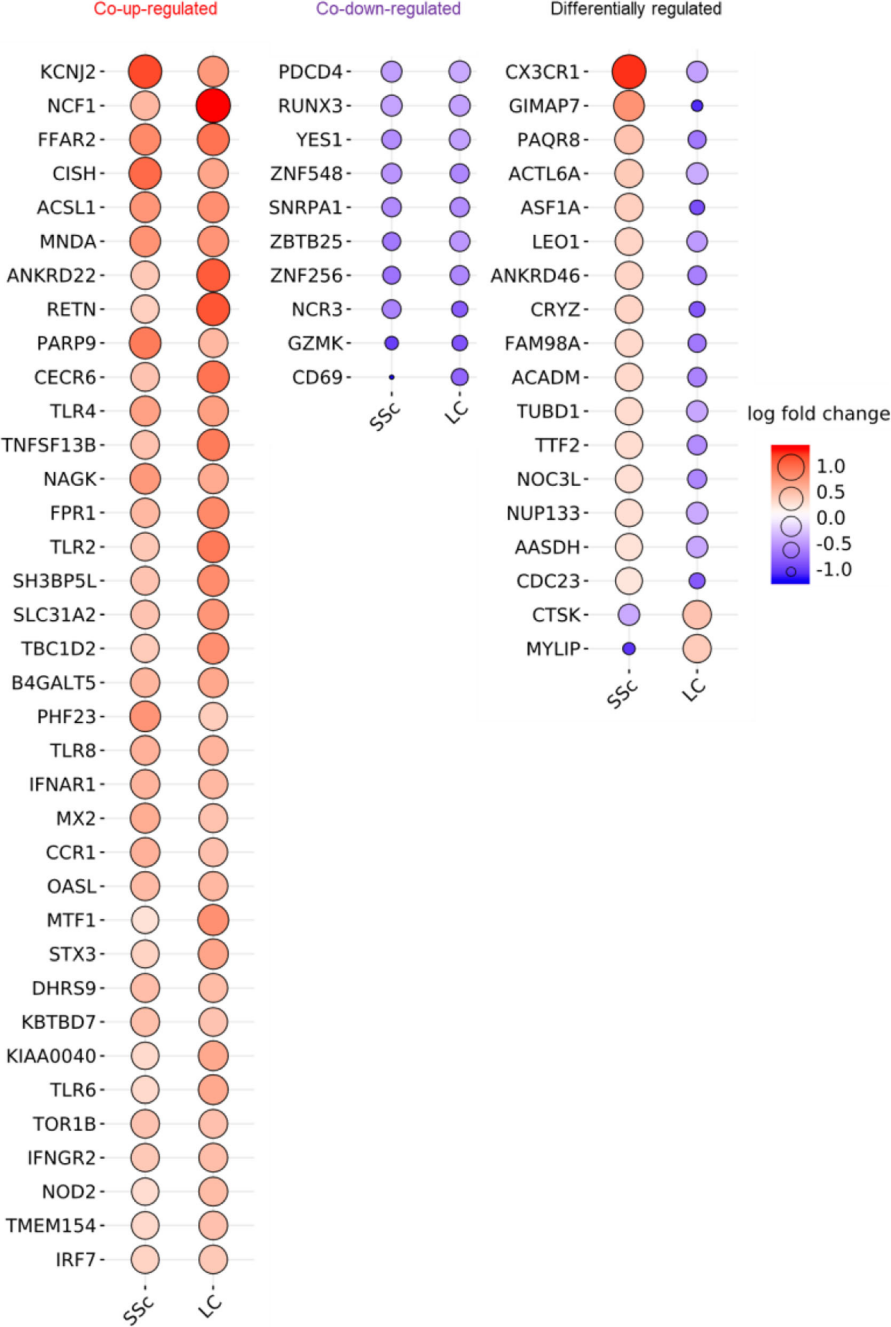
Overview of DEGs present in both systemic sclerosis and lung cancer. Red and large circles indicate up-regulation, purple and small circles indicate down-regulation.

The acquisition of common DEGs in all patients laid a solid foundation for our analysis of the differences and links between the two diseases. Unlike the above analysis revealing commonalities of all patients with the same disease, not only the same regulatory trend but also the distinction between systemic sclerosis and lung cancer attracted our great interest. We investigated 299 DEGs in systemic sclerosis and 1644 DEGs in lung cancer. Finally found 64 overlapping DEGs, which accounted for 22.07 and 4.02% of the total DEGs in systemic sclerosis and lung cancer. It indicated that there is a small proportion of overlapping DEGs between the two diseases. Among the overlapping DEGs, 36 co-upregulated DEGs, 10 co-downregulated DEGs, and 18 DEGs with opposite regulatory trends were identified, and details of the changes were displayed (Fig. 5b and 6). Co-upregulated DEGs accounted for the largest proportion, while a few genes were co-downregulated. Most of the DEGs with opposite regulatory trends were upregulated in systemic sclerosis but downregulated in lung cancer.

### Gene expression overlap shared by both systemic sclerosis and lung cancer

**Fig. 7:**
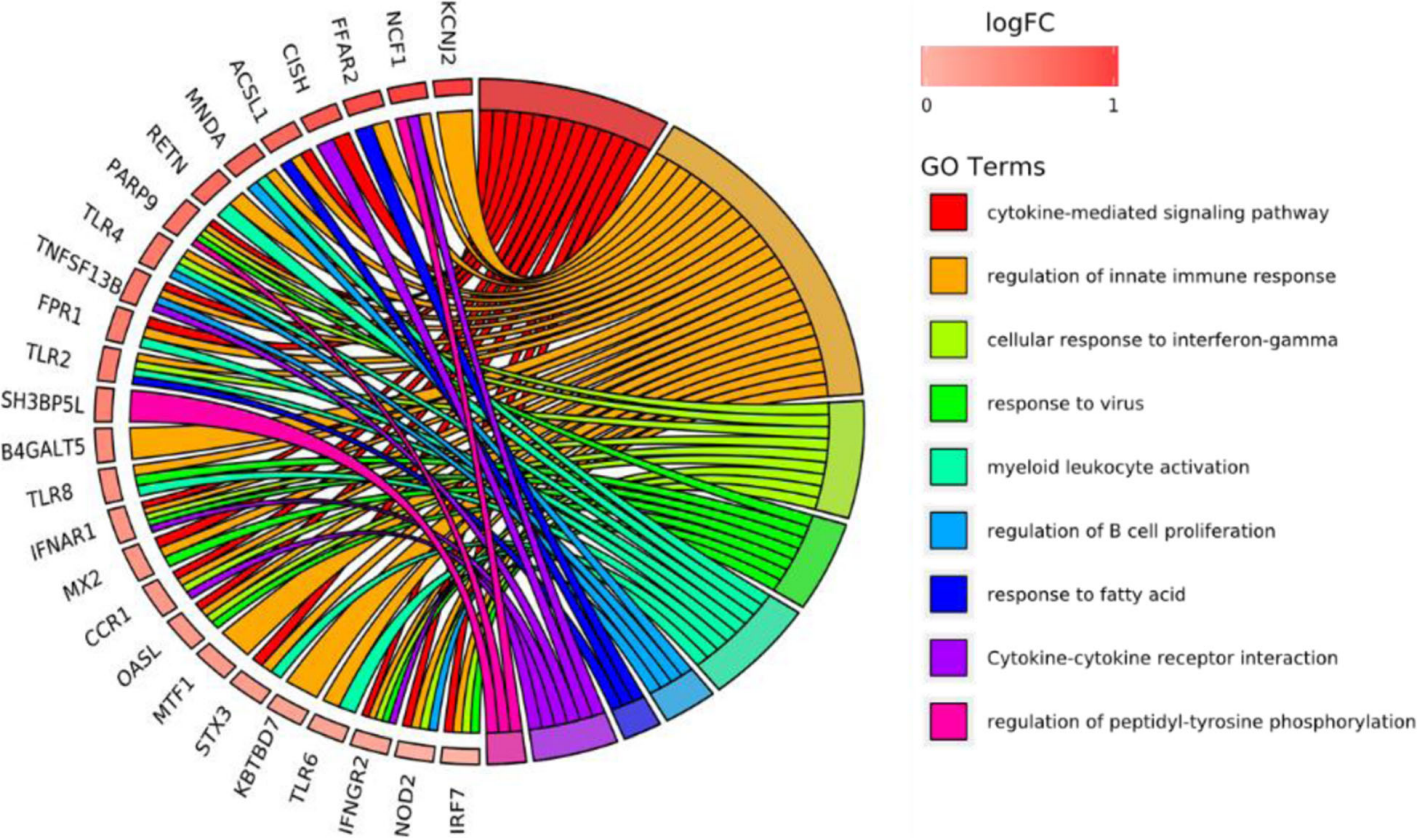
Distribution of co-upregulated DEGs in systemic sclerosis and lung cancer for main biological processes, ranked by fold change of genes

Considering the above fact that co-up-regulated and co-down-regulated genes were dominant (72%) in the above comparative analysis, we subsequently focused on the gene expression overlap between systemic sclerosis and lung cancer. First, we performed a functional enrichment analysis to explore the same intrinsic mechanisms of both diseases. Through the analysis of 36 co-upregulated DEGs distribution, these DEGs were found to come from different pathways (Fig. 7). Most of the DEGs (24/36) were related to regulation of innate immune response (GO: 0045088). Except for CISH, the top 12 DEGs were all associated with innate immune response showing its critical role in fighting both diseases. Secondly, these DEGs are mainly mediated by a series of cytokine signaling pathway (GO: 0019221), including regulation of cytokine secretion (GO: 0050707) and cytokine secretion involved in immune response (GO: 0002374). Other GO terms include cellular response to interferon-gamma, response to virus, myeloid leukocyte activation, regulation of B cell proliferation, response to fatty acid, cytokine-cytokine receptor interaction, and regulation of peptidyl-tyrosine phosphorylation. These terms were characterized by antiviral, immunomodulatory, and anti-tumor properties.

**Fig. 8:**
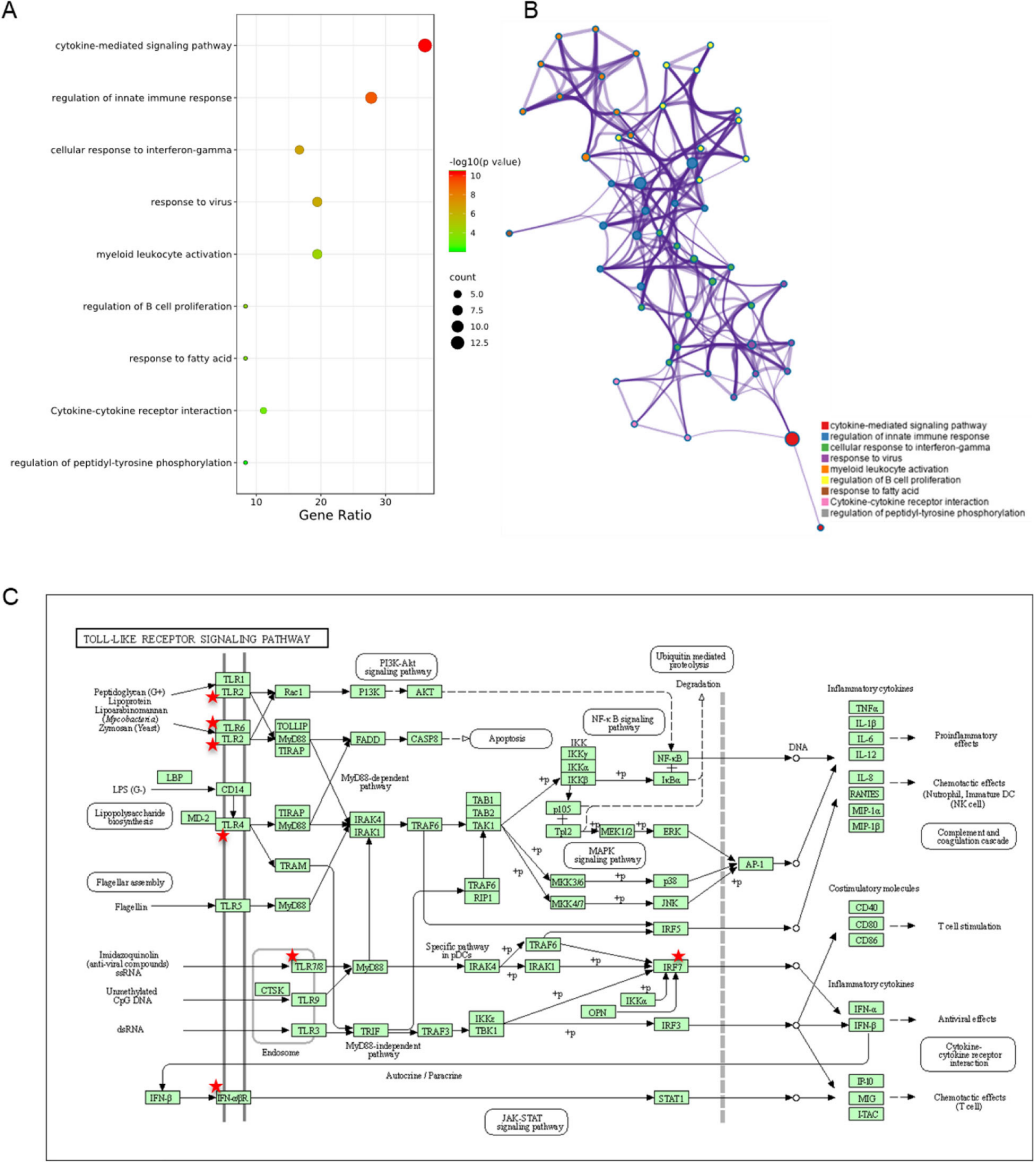
**a** Gene ontology enrichment analysis of co-upregulated DEGs in both systemic sclerosis and lung cancer. **b** Integrated network analysis reveals a core position of innate immunity among all pathways. **c** KEGG pathway mapping of co-upregulated DEGs in both systemic sclerosis and lung cancer

Further enrichment analysis showed that the co-up-regulated DEGs are mainly enriched in cytokine-mediated signaling pathway, regulation of innate immune response, cellular response to interferon-gamma and response to virus (Fig. 8a). Among them, innate immunity is located in the core position, which connects regulation of B cell proliferation, myeloid leukocyte activation and cellular response to interferon-gamma (Fig. 8b). These results indicate that cytokine-mediated signaling pathway and regulation of innate immune response were the key biological processes in both systemic sclerosis and lung cancer compared with healthy controls. The co-upregulated DEGs were involved in a series of cytokine-mediated signaling pathways, affecting various receptors on the cell surface. Through KEGG pathway analysis, we found that TLR2, TLR4, TLR6, TLR8, IFNAR1, IFNGR2 and IRF7 in toll-like receptors signaling pathway were significantly upregulated in both diseases, thereby affecting the innate immunity of cells (Fig. 8c).

**Fig. 9:**
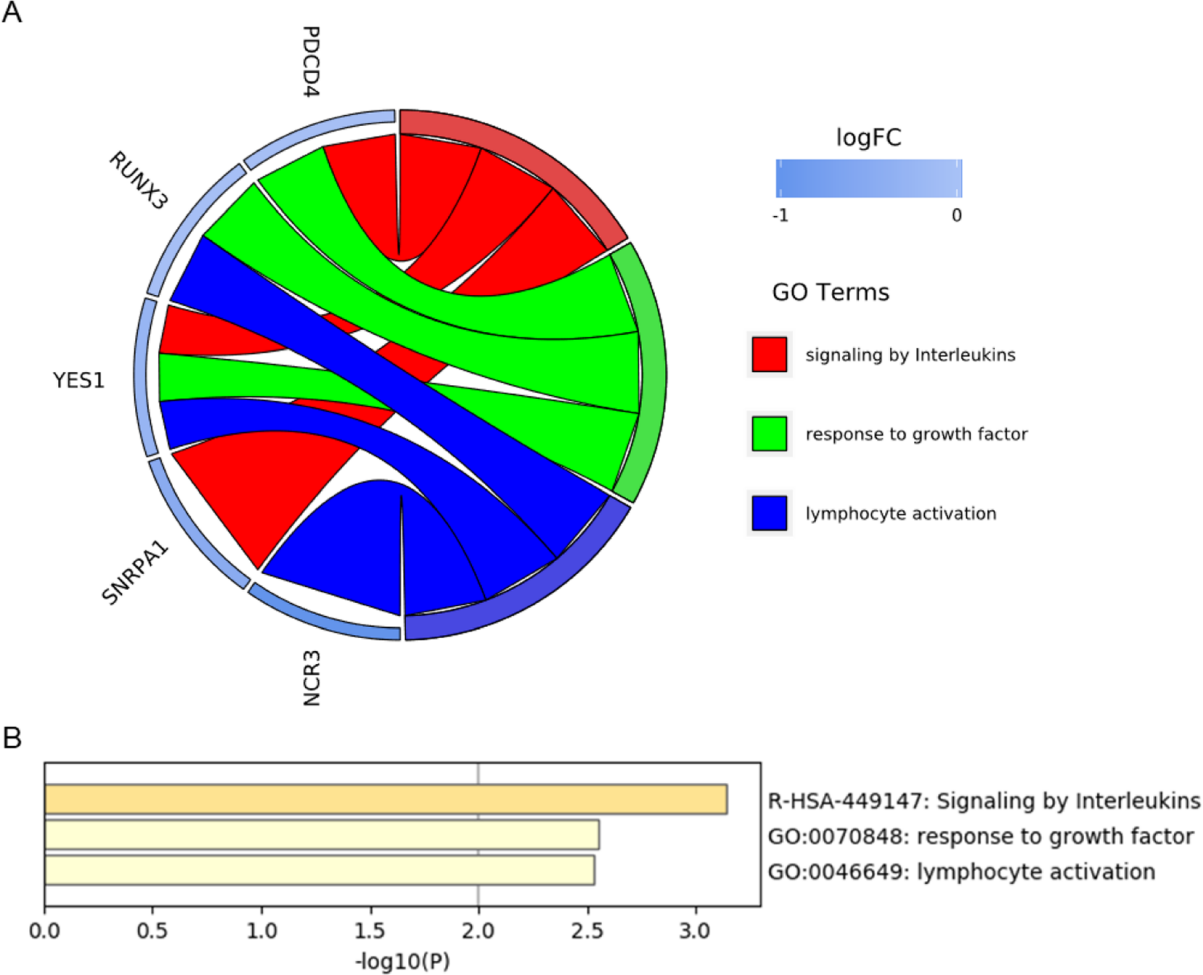
Distribution of co-down-regulated DEGs in systemic sclerosis and lung cancer for GO terms. **b** Significant pathway enrichment of DEGs co-down-regulated in both systemic sclerosis and lung cancer

On the other hand, GO enrichment analysis of 10 co-down-regulated DEGs yielded three terms: signaling by interleukins, response to growth factor and lymphocyte activation, each term contained three DEGs (Fig. 9a), the all three being significant (Fig. 9b). The down-regulation of signaling by interleukins indicates that the communication between lymphocytes may be weakened, which may influencethe function of lymphocytes. Despite lower significance compared with signaling by interleukins, reduced growth factor and lymphocyte activation also weakened the function of immune system. The down-regulation of these terms may affect the maturation, proliferation and activation of immune cells.

### Potential differential pathways between systemic sclerosis and lung cancer

**Fig. 10:**
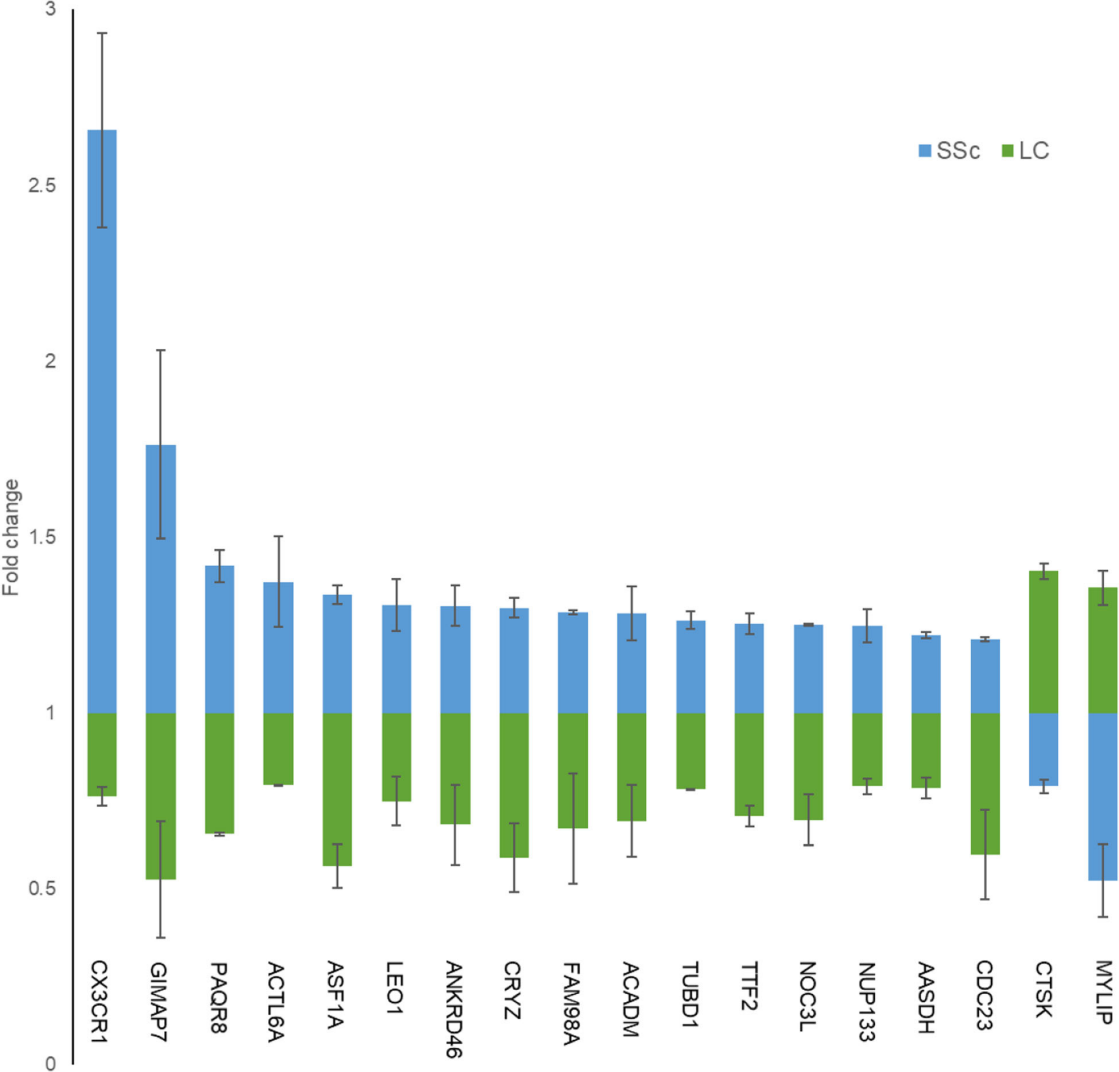
KEGG analysis revealed associations between genes or pathways

To identify the core gene expression differences between systemic sclerosis and lung cancer, we compared the most important DEGs with the opposite trends. The expression difference of DEGs in the two diseases was visualized by the total lengths of blue and green columns (Fig. 10). Most genes were up-regulated in systemic sclerosis and down-regulated in lung cancer, except for CTSK and MYLIP. The top DEGs with the greatest differences were GIMAP7, CX3CR1, MYLIP and ASF1A. Among them, GIMAP7 was the most significantly downregulated in lung cancer, CX3CR1 was the most upregulated and MYLIP was the most downregulated in systemic sclerosis.

**Fig. 11:**
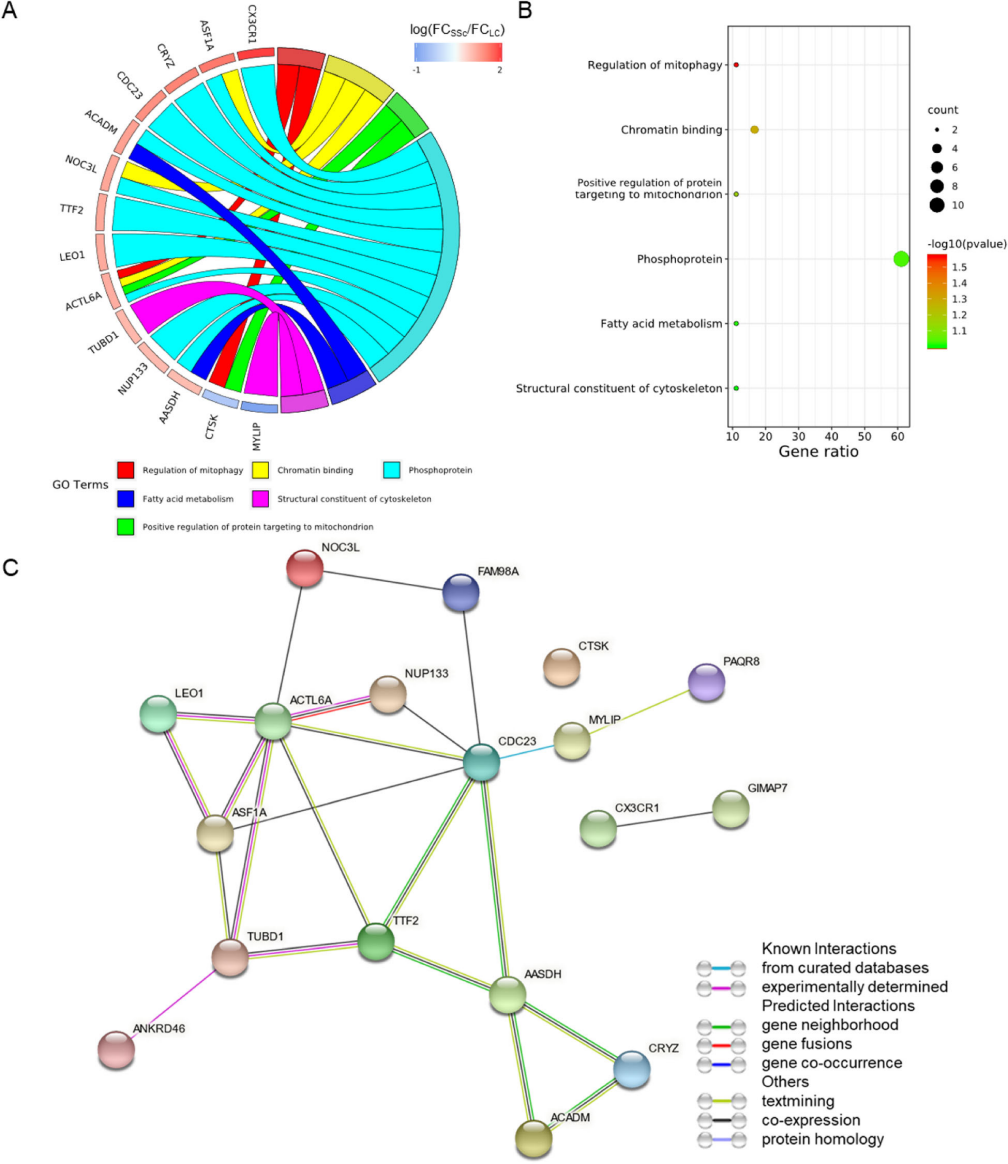
Biological process distribution (**a**), enrichment analysis (**b**) and Protein-protein interaction networks (**c**) of DEGs with opposite trends in systemic sclerosis and lung cancer

Therefore, we performed an enrichment analysis to further identify the potential mechanisms of these associations. Using databases including KEGG, GO, and BioCarta, six terms were enriched. The signaling pathways of DEGs were primarily enriched in regulation of mitophagy (*P* = 0.027), followed by chromatin binding, positive regulation of protein targeting to the mitochondrion, phosphoprotein and fatty acid metabolism (Fig. 11a and b). The DEG list was imported into STRING database (http://string-db.org) to explore their interrelationships and calculate the characteristics of the network (Fig. 11c), with a total of 18 DEGs. Except that CTSK is isolated, a complex network was constructed, in which MYLIP and CDC23 had the highest confidence.

**Fig. 12:**
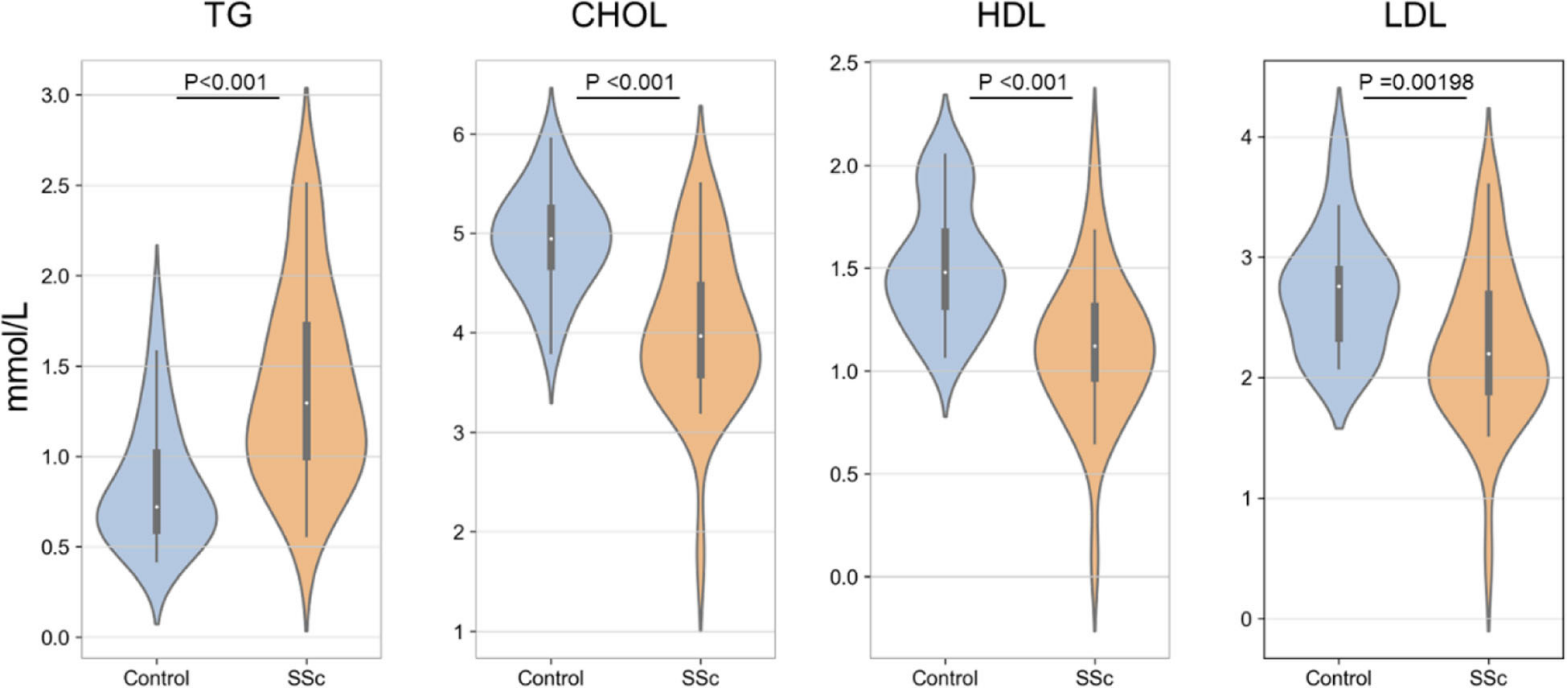
The concentration of triglycerides, total cholesterol, HDL-C, and LDL-C in blood of systemic sclerosis (SSc) patients and healthy controls

MYLIP is involved in low density lipoprotein receptor (LDLR) degradation, also considering that the co-upregulated DEGs ACADM, NOC3L and AASDH are involved in fatty acid metabolism. This implies that fatty acid metabolism may be associated with the disease process. We retrospectively analyzed lipid data from 32 patients with systemic sclerosis, healthy controls were individually matched according to sex and age. The concentrations of triglycerides, total cholesterol, high density lipoprotein cholesterol (HDL-C), and low density lipoprotein cholesterol (LDL-C) are shown, and overall levels in healthy individuals are within normal reference ranges (Fig. 12).

## Discussion

Lung cancer has the highest morbidity and mortality among all cancers [1, 12], and its early diagnosis has significant challenges. Pulmonary symptoms of various diseases interfere with the diagnosis of lung cancer, especially some autoimmune diseases that can increase the incidence of lung cancer. Blood-based biomarkers would be very useful for early diagnosis of lung cancer. Disease-related gene expression characteristics in peripheral blood mononuclear cells (PBMC) have been described in several types of cancer. However, RNA-stabilized whole blood technology could be more applicable and powerful in the clinic. We conducted a comprehensive study of immune-related genes in systemic sclerosis and lung cancer based on gene expression profiling in whole blood. Our results show that systemic sclerosis and lung cancer share a common trend of change in innate immunity and cell cycle regulation, while in some pathways there are opposite characteristics. These results improve our understanding of the different strategies of the immune system to combat lung cancer and systemic sclerosis, and prompt further development of biomarkers based on gene expression in peripheral blood for early detection of lung diseases.

Pulmonary manifestations are known to occur in all autoimmune diseases that have standardized incidence ratios greater than 2.0, indicating that autoimmune process can lead to lung cancer susceptibility [8]. Systemic sclerosis is one of the connective tissue diseases with the highest mortality rate [11, 22], and it has recently been reported that patients with systemic sclerosis have an increased risk of lung cancer [23, 24], but the intrinsic gene connection is unclear. Our results reveal that genes related to innate immune response and response to virus were co-upregulated significantly in patients with systemic sclerosis and lung cancer. And the expression of genes related to negative regulation of cell cycle and transcription factor activity decreased considerably. In contrast, the expression of genes related to mitophagy regulation and chromatin binding showed a clear opposite trend. Although the fold changes of DEGs just ranged from 0.46 to 2.66, these changes were evident and stable in all patients in this study, providing some reliable support for the intrinsic association between systemic sclerosis and lung cancer. It has been previously reported that certain genes may be critical in the pathogenesis of systemic sclerosis (chemokine CCL2 and CXCL4 [25]) and lung cancer (including EGFR and HER2 [26, 27]), but our results are more convincing in terms of reliability and universality owing to the large sample size. It has also been hypothesized that certain treatments for systemic sclerosis (e.g., immunosuppressants) may lead to a decline in immunity, which in turn increases the cancer risk [28]. However, this cannot explain the higher degree of pulmonary fibrosis in lung cancer patients. In summary, this evidence provides some support for the potential immune system association between systemic sclerosis and lung cancer.

In a recent study of the demographic and clinicopathological characteristics of lung cancer in patients with systemic sclerosis, 1.4% patients developed systemic sclerosis before the onset of lung cancer (~ 13 years averagely) [29]. The distribution of histological types of cancer is similar to that of the general population, most of which are adenocarcinomas, followed by squamous and small cell carcinomas. This is consistent with the reported histological pattern of systemic sclerosis-associated lung cancer [30]. Notably, none of these patients ever smoked, suggesting that systemic sclerosis is an independent risk factor for lung cancer. The study confirmed a lack of common cancer driver for gene mutations in these patients, further supporting the possible role of chronic autoimmune inflammation in carcinogenesis of systemic sclerosis-related lung cancer. However, the study did not reveal changes in disease-related genes.

Although innate immunity is enhanced in both diseases, its effect on the diseases is complicated. Excessive innate immunity may negatively affect systemic sclerosis, while it may contribute to the elimination of cancer cells. On the other hand, among the 10 significantly co-downregulated genes, PDCD4 and YES1 are related to apoptosis [31, 32], while NCR3 and CD69 are related to cell killing [33, 34]. It can be speculated that the loss of relevant roles is one of the common pathogenic causes. Both PDCD4 and RUNX3 are tumor suppressor genes [35–37], and their co-downregulation may be one of the causes of lung cancer.

Among the counter-regulated DEGs, ACTL6A and CTSK belonged to mitophagy regulation, ASF1A, NOC3L and ACTL6A belonged to chromatin binding. Although ACTL6A is involved in both pathways, in fact it is a component of chromatin remodeling complexes and is indirectly involved in transcriptional activation or repression [38], the significance remains to be determined. CTSK displays potent endoprotease activity against fibrinogen [39], plays an important role in extracellular matrix degradation, its counter-regulation in lung cancer and systemic sclerosis may lead to different effects on the extracellular matrix. NOC3L is a homolog of nucleolar complex protein 3, may be required for adipogenesis. ASF1A functions as a histone chaperone in nucleosome assembly and disassembly [40]. As chromatin binding proteins, NOC3L and ASF1A may be indirectly related to diseases.

GIMAP (GTPase of the immunity-associated protein) gene family encodes unique GTPases, most of which functions are unknown. Abnormal expression of GIMAP family members in cancer tissues has also been reported in non-small cell lung cancer, and qPCR analysis showed downregulation of GIMAP6 and GIMAP8 in lung cancer tissues [41]. Another report showed that downregulation of GIMAP7 at both protein and mRNA levels observed in serum and tissue samples of oral cancer patients may imply that GIMAP7 has an anticancer effect [42]. Downregulation of GIMAP gene may regulate immune cell viability or development affecting cancer progression [43], however, high expression of GIMAP7 in systemic sclerosis has not been reported, which needs further exploration.

The important role of chemokine receptors in disease pathogenesis has caused much attention. In peripheral blood, chemokine receptor CX3CR1 is present on the cell membrane of monocytes and leukocytes [44, 45], it is not only involved in cell chemotaxis, but is also related to cell adhesion [46]. The arrival of inflammatory cells from the peripheral circulation to the site of inflammation is a dynamic, multistep process in which CX3CR1 plays a crucial role [47]. An increased level of CX3CR1 in systemic sclerosis may help to recruit immune cells to the site of inflammation, giving CX3CR1 an important role in the pathogenesis and development of inflammatory response in autoimmune diseases. Animal experiments and preclinical studies with blocking CX3CR1 signaling showed good anti-inflammatory effects. However, a decreased level of CX3CR1 in lung cancer may be related to immune weakness and attenuate cell adhesion, which may be associated with cell metastasis. The co-expression characteristics of CX3CR1 and GIMAP7 may play an overlapping role in the occurrence and development of both diseases. Anti-silencing function protein 1 homolog A (ASF1A) is an abundant histone remodeling chaperone in meiosis phase 2. Recent studies uncover Asf1a as a tumor-intrinsic suppressor of immune checkpoint blockade through suppression of GM-CSF expression [48]. Functional analysis of another study showed that the interaction between ASF1A and E2 ubiquitin-binding enzymes was associated with tumorigenesis [49].

Myosin regulatory light chain interacting protein (MYLIP, also named IDOL) is an important ubiquitin E3 ligase, which mediates low density lipoprotein receptor (LDLR) degradation through ubiquitination reaction and affect blood lipid levels [50]. Previous reports indicated the LDLR-related protein (LRP1B) was discovered as a putative tumor suppressor and frequently inactivated in lung cancer cells [51, 52]. It may be related to the upregulation of protein MYLIP which mediates LDLR degradation. In this study, dyslipidemia was found to be fairly common in systemic sclerosis patients, with an average increase in triglycerides of 64.4% (*P* = 0.012) and a predominant decrease (17.0–26.7%) in total cholesterol, HDL-C and LDL-C, which may be associated with changes of lipoprotein receptor. A recent study in Cell Reports revealed that short chain fatty acids in the gut and blood can regulate macrophages to suppress bacterial infection by activating free fatty acid receptor 2 (FFAR2) [53]. Therefore, the relationship between fat metabolism and immune regulation requires more investigation. In addition, the increased level of chain fatty acid CoA ligase 1 (ACSL1) may contribute to abnormal lipid level.

## Conclusion

Our study detected the association and differences in immune-related genes between systemic sclerosis and lung cancer using multiple cohorts based on different populations, which will help us understand the relationship of autoimmune diseases and cancers, as well as the mechanisms by which the immune system responds to different diseases. The DEG overlap between systemic sclerosis and lung cancer may partly explain the clinical lung phenotypic association, and the counter-regulated DEGs may provide potential molecular diagnostic markers and biological clues for the two diseases. However, further studies are needed to reveal how the identified gene signatures relate to the pathogenesis of both diseases.

## Supplementary Information

**Additional file 1: Table S1.** Data of 61 patients with systemic sclerosis, 70 patients with lung cancer and 224 normal healthy individuals. Underlined accessions are removed by principal component analysis. **Fig. S1**. Hierarchical clustering heatmap of top-ranked DEGs screened from systemic sclerosis (Group I and Group II) and lung cancer (Group III and Group IV).

## Data Availability

All data of this study are included in this published article.
