# Supplementary material for: Integrative genomic expression analysis reveals stable differences between lung cancer and systemic sclerosis
Source: BMC Cancer. 2021 Mar 10;21:259. doi: 10.1186/s12885-021-07959-6 (PMC7944918; doi:10.1186/s12885-021-07959-6)
Supplement: Supplementary file 1 — Additional file 1: Table S1. Data of 61 patients with systemic sclerosis, 70 patients with lung cancer and 224 normal healthy individuals. Underlined accessions are removed by principal component analysis. Fig. S1. Hierarchical clustering heatmap of top-ranked DEGs screened from systemic sclerosis (Group I and Group II) and lung cancer (Group III and Group IV). [file 12885_2021_7959_MOESM1_ESM.pdf]

Integrative genomic expression analysis reveals stable differences between lung cancer and systemic sclerosis

Heng Li[1,2], Liping Ding[1], Xiaoping Hong[1], Yulan Chen[1], Rui Liao[1], Tingting Wang[1,2], Shuhui Meng[1], Zhenyou Jiang[3,*], Dongzhou Liu[1,*]

[1]Department of Rheumatology and Immunology, Shenzhen People's Hospital, The Second Clinical Medical College of Jinan University, Shenzhen 518020, China

[2]Integrated Chinese and Western Medicine Postdoctoral research station, Jinan University, Guangzhou 510632, China

[3]Department of Microbiology and Immunology, School of Medicine, Jinan University, Guangzhou, China

*Correspondence: liu_dz2001@sina.com (Dongzhou Liu) and tjzhy@jnu.edu.cn (Zhenyou Jiang)

Table S1 Data of 61 patients with systemic sclerosis, 70 patients with lung cancer and 224 normal healthy individuals. Underlined accessions are removed by principal component analysis.

Group		Accession	Platform
I	SSc (n=20)	GSM556413, GSM556414, GSM556415, GSM556416, GSM556417, GSM556418, GSM556419, GSM556420, GSM556421, GSM556422, GSM556423, GSM556424, GSM556425, GSM556426, GSM556427, GSM556428, GSM556429, GSM556430, GSM556431, GSM556432	GPL570
	Normal (n=10)	GSM556441, GSM556442, GSM556443, GSM556444, GSM556445, GSM556446, GSM556447, GSM556448, GSM556449, GSM556450	GPL570
II	SSc (n=69)	GSM827736, GSM827737, GSM827738, GSM827739, GSM827740, GSM827741, GSM827742, GSM827743, GSM827744, GSM827745, GSM827746, GSM827747, GSM827748, GSM827749, GSM827750, GSM827751, GSM827752, GSM827753, GSM827754, GSM827755, GSM827756, GSM827757, GSM827758, GSM827759, GSM827760, GSM827761, GSM827762, GSM827763, GSM827764, GSM827765, GSM827766, GSM827767, GSM827768, GSM827769, GSM827770, GSM827771, GSM827772, GSM827773, GSM827774, GSM827775, GSM827776, GSM827777, GSM827778, GSM827779, GSM827780, GSM827781, GSM827782, GSM827783, GSM827784, GSM827785, GSM827786, GSM827787, GSM827788, GSM827789, GSM827790, GSM827791, GSM827792, GSM827793, GSM827794, GSM827795, GSM827796, GSM827797, GSM827798, GSM827799, GSM827800, GSM827801, GSM827802, GSM827803, GSM827804	GPL6947
	Normal (n=41)	GSM827665, GSM827666, GSM827667, GSM827668, GSM827669, GSM827670, GSM827671, GSM827672, GSM827673, GSM827674, GSM827675, GSM827676, GSM827677, GSM827678,	GPL6947

		GSM827679, GSM827680, GSM827681, GSM827682, GSM827683, GSM827684, GSM827685, GSM827686, GSM827687, GSM827688, GSM827689, GSM827690, GSM827691, GSM827692, GSM827693, GSM827694, GSM827695, GSM827696, GSM827697, GSM827698, GSM827699, GSM827700, GSM827701, GSM827702, GSM827703, GSM827704, GSM827705	
	LC (n=16)	GSM1050776, GSM1050795, GSM1050811, GSM1050814, GSM1050817, GSM1050818, GSM1050840, GSM1050855, GSM1050929, GSM1050936, GSM1050958, GSM1050961, GSM1050967, GSM1050980, GSM1050993, GSM1051018	GPL10558
III	Normal (n=113)	GSM1050741, GSM1050742, GSM1050743, GSM1050744, GSM1050745, GSM1050746, GSM1050747, GSM1050749, GSM1050750, GSM1050751, GSM1050752, GSM1050753, GSM1050755, GSM1050756, GSM1050757, GSM1050764, GSM1050765, GSM1050768, GSM1050769, GSM1050770, GSM1050771, GSM1050772, GSM1050773, GSM1050781, GSM1050782, GSM1050785, GSM1050786, GSM1050787, GSM1050788, GSM1050794, GSM1050796, GSM1050799, GSM1050800, GSM1050803, GSM1050804, GSM1050805, GSM1050806, GSM1050808, GSM1050809, GSM1050810, GSM1050812, GSM1050815, GSM1050819, GSM1050820, GSM1050821, GSM1050823, GSM1050824, GSM1050825, GSM1050827, GSM1050828, GSM1050829, GSM1050831, GSM1050832, GSM1050833, GSM1050835, GSM1050837, GSM1050838, GSM1050839, GSM1050841, GSM1050842, GSM1050844, GSM1050846, GSM1050847, GSM1050849, GSM1050850, GSM1050851, GSM1050852, GSM1050853, GSM1050854, GSM1050856, GSM1050858, GSM1050859, GSM1050860, GSM1050861, GSM1050862, GSM1050928, GSM1050932, GSM1050935, GSM1050937, GSM1050938, GSM1050939, GSM1050940, GSM1050941, GSM1050947, GSM1050954, GSM1050956, GSM1050957, GSM1050959, GSM1050962, GSM1050964,	GPL10558

		GSM1050968, GSM1050972, GSM1050978, GSM1050981, GSM1050984, GSM1050987, GSM1050989, GSM1050991, GSM1050992, GSM1050995, GSM1050997, GSM1050998, GSM1050999, GSM1051000, GSM1051004, GSM1051010, GSM1051011, GSM1051012, GSM1051013, GSM1051015, GSM1051016, GSM1051017, GSM1051020	
IV	LC (n=54)	GSM320318, GSM320319, GSM320320, GSM320321, GSM320322, GSM320323, GSM320324, GSM320325, GSM320326, GSM320327, GSM320328, GSM320329, GSM320330, GSM320331, GSM320332, GSM320333, GSM320334, GSM320335, GSM320336, GSM320337, GSM320338, GSM320339, GSM320340, GSM320341, GSM320342, GSM320343, GSM320344, GSM320345, GSM320346, GSM320347, GSM320348, GSM320349, GSM320350, GSM320351, GSM320352, GSM320353, GSM320354, GSM320355, GSM320356, GSM320357, GSM320358, GSM320359, GSM320387, GSM320388, GSM320389, GSM320390, GSM320391, GSM320393, GSM320394, GSM320396, GSM320397, GSM320398, GSM320400, GSM320401	GPL6102
	Normal (n=32)	GSM320361, GSM320362, GSM320363, GSM320365, GSM320366, GSM320367, GSM320369, GSM320370, GSM320371, GSM320372, GSM320373, GSM320375, GSM320376, GSM320377, GSM320379, GSM320380, GSM320382, GSM320383, GSM320385, GSM320404, GSM320405, GSM320407, GSM320408, GSM320410, GSM320411, GSM320413, GSM320415, GSM320416, GSM320417, GSM320418, GSM320419, GSM320420	GPL6102

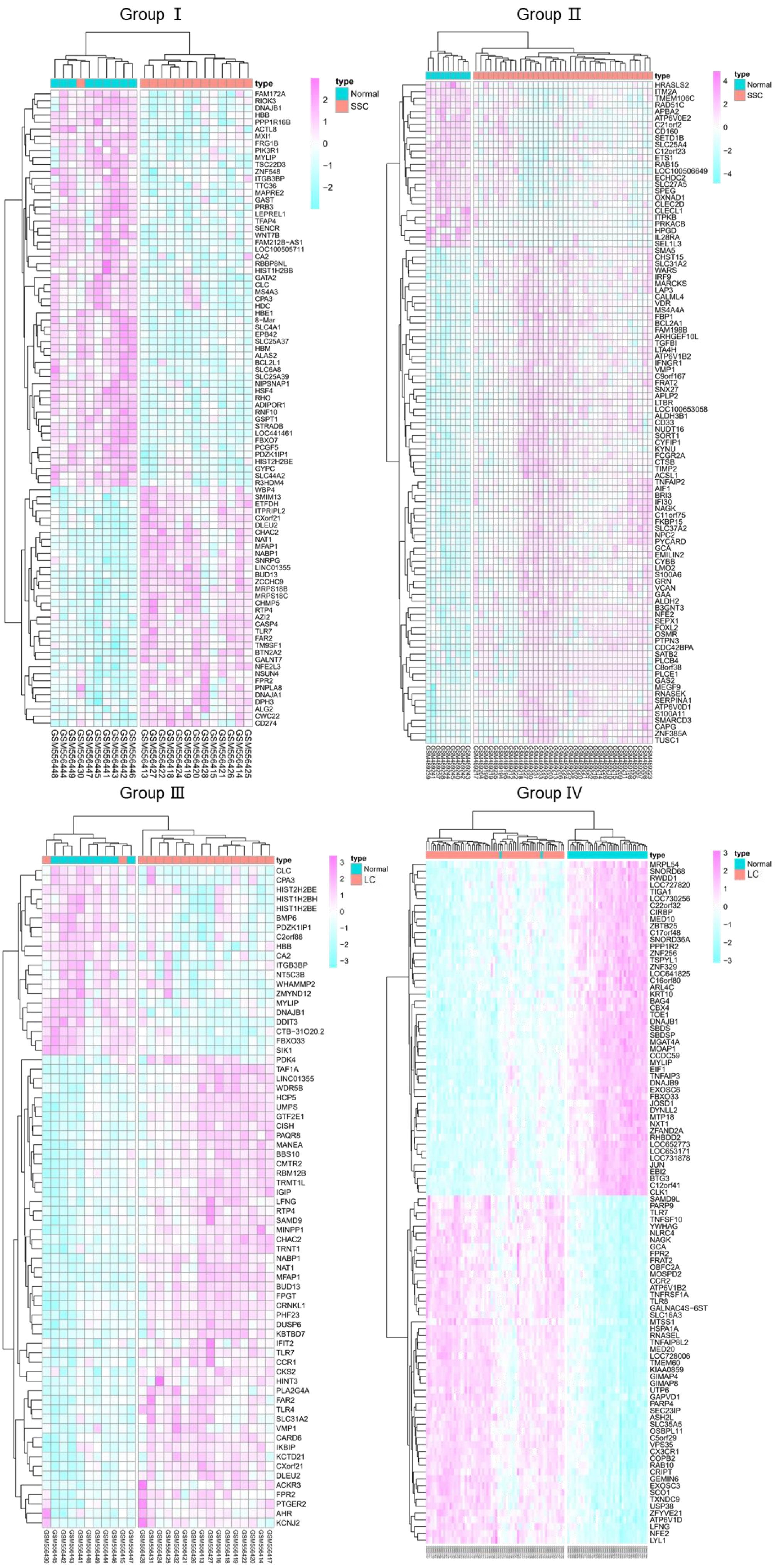

Group Ⅰ
type
Normal
SSC
Group Ⅱ
type
Normal
SSC
Group Ⅲ
type
Normal
LC
Group Ⅳ
type
Normal
LC

Fig. S1. Hierarchical clustering heatmap of top-ranked DEGs screened from systemic sclerosis (Group I and Group II) and lung cancer (Group III and Group IV).
